# Stratification Requirements for Seed Dormancy Alleviation in a Wetland Weed

**DOI:** 10.1371/journal.pone.0071457

**Published:** 2013-09-05

**Authors:** Louis G. Boddy, Kent J. Bradford, Albert J. Fischer

**Affiliations:** 1 Plant Science Department, Marrone Bio Innovations, Davis, California, United States of America; 2 Department of Plant Sciences, University of California Davis, Davis, California,United States of America; University College London, United Kingdom

## Abstract

*Echinochloaoryzicola*(syn.*E. phyllopogon*) is an exotic weed of California rice paddies that has evolved resistance to multiple herbicides. Elimination of seedlingsthroughcertain weed control methods can limit the spread of this weed, but is contingent on accurate predictions of germination and emergence timing, which are influenced by seed dormancy levels.In summer annuals, dormancy can often be relieved through stratification, a period of prolonged exposure to cold and moist conditions.We used population-based threshold models to quantify the effects of stratification on seed germination of four *E. Oryzicola* populations at a range of water potential (*Ψ*) and oxygen levels. We also determined how stratification temperatures, moisture levels and durations contributed to dormancy release. Stratification released dormancy by decreasing base *Ψ* and hydrotimerequired for germination and by eliminating any germination sensitivity to oxygen. Stratification also increased average germination rates (GR), which were used as a proxy for relative dormancy levels. Alternating temperatures nearly doubled GR in all populations, indicating that seeds could be partially dormant despite achieving high final germination percentages. Stratification at *Ψ* = 0 MPa increased GR compared to stratification at lower water potentials, demonstrating that *Ψ* contributed to regulating dormancy release. Maximum GR occurred after 2-4 weeks of stratification at 0 MPa; GR were often more rapid for herbicide-resistant than for herbicide-susceptible seeds, implying greater dormancy in the latter. Manipulation of field conditions to promote dormancy alleviation of *E. oryzicola* seeds might improve the rate and uniformity of germination for seed bank depletion through seedling weed control. Our results suggest field soil saturation in winter would contribute towards *E. oryzicola* dormancy release and decrease the time to seedling emergence.

## Introduction

The temperate region summer annual weed *Echinochloaoryzicola*Vasing. (syn. *E.phyllopogon*Stapf ex Kossenko) is a morphological mimic of rice (*Oryza sativa*) that can germinate and initiate shoot growth under hypoxia in flooded paddies [1] and causes up to 50% rice yield losses in California if not controlled [2], [3]. Decades of heavy reliance on herbicides for *E. oryzicola* control [3] have resulted in the widespread occurrence of populations with simultaneous resistance to most available grass herbicidesfor selective use in rice [4]–[7].Successful control of herbicide-resistant *E. oryzicola* now hinges on maximizing weed seedling recruitment in order to eliminate such seedlings prior to planting the crop [8], [9].The stale seedbed approach entails recruiting and treating weeds prior to planting rice with a mechanical method or a broad-spectrum herbicide for which resistance does not exist in these weeds [9], [10]. The effectiveness of this approach would be optimized if the timing of weed seedling emergence under varying temperatures and irrigation regimes could be accurately predicted and if the conditions for maximizing emergence rate and synchrony could be identified [11].

Population-based threshold models (PBTM) have been developed to describe germination responses to temperature, water potential [12] and oxygen [13], and have been used to predict crop seedling emergence [14], [15]. For non-dormant *E. oryzicola* seed, the PBTM approach predicted with useful accuracy the germination responses of seeds to shifting temperature and water availability and their subsequent emergence from field soils [16]. However, *Poaceae* seeds typically possess non-deep physiological dormancy (NDPD), which indicates that seed dormancy release and increases in germination rates (speed)vary along a continuum of time and environmental conditions [17], [18]. NDPD may be released by stratification, after-ripening, scarification, excision of the embryo or addition of gibberellin [17] and by various environmental signals including light, fluctuating temperatures and soil nitrate [19]. In addition, the environmental requirements for dormancy alleviationare often population- rather than species-specific [20]–[22], thus requiring analysis at the population level. While non-dormant seeds of selected herbicide-resistant (R) and herbicide-susceptible (S) populations of *E. oryzicola* germinated similarly [16], information on differences in seed dormancy between R and S populations is lacking. Herbicide-resistant *E. oryzicola* populations trace their origin to a single introduced biotype dispersed throughout California rice fields [23] suggestingthat R populations may respond similarly to environmental variables affecting germination and dormancy.

As in many summer annual species with NDPD [24], innate dormancy of *E. oryzicola* seed populations that emerge in spring [1] is alleviated by cold stratification when exposed to a period of moisture at wintertime temperatures in California. Thus,hydration and dark storage at 3°C alleviated dormancy of most seeds in this species [16]. In California, yearly wintertime variation in field temperatures may be less than year-to-year variation in moisture levels, which may range from sporadic rain to prolonged periods of flooding [25]. Adaptation to these conditions would suggest that stratification moisture levels may influence the magnitude of *E. oryzicola* seed dormancy release and that dormancy levels could perhaps be manipulated using wintertime irrigation to increase the rate of springtime germination and weed seedling recruitment [26]. The median base water potential estimated using hydrotime germination models is often a measure of the relative dormancy status of a seed population [12], and because dormancy removal enables *E. oryzicola* seeds to transition from aerobic respiration to anaerobic alcoholic fermentation [1], oxygen-time germination models [13] might also provide a means of assessing dormancy levels in seeds of this species.

To understand the environmental requirements for *E. oryzicola* seed dormancy alleviation, we sought here to: 1) quantify stratification effects upon germinationof seeds of R and S populations of *E. Oryzicola* across a range of moisture and oxygen levels; and 2) ascertain the relative contributions of alternating temperatures and of stratification temperature, water potential and duration towards dormancy release in R and S *E. oryzicola* populations. This knowledge will contribute towards the accuracy of germination-based predictions of seedling emergence as affected by the dormancy status of the seed and thus improve the timing and efficacy of weed control programs.

## Materials and Methods

### Plant material and general experimental conditions

*E. oryzicola* seeds (spikelets) of four populations (CR, HR, KS and SW) representing the range of phenotypic variability previously reported in California [23] were mass collected from Sacramento Valley, California, rice fields (with consent of field owners) between 1997 and 2002 [16] and used in all experiments of this study. Populations CR and HR were subsequently classified as herbicide-susceptible (S) and populations KS and SW as herbicide-resistant (R) [16], [27]. In the summers of 2007 and 2009, 38 plants from each population were placed in separate greenhouses for seed multiplication at the University of California, Davis. Plants were grown in 2-L pots filled with soil placed in flooded basins under conditions set to approximate mid-springtime field conditions in the Sacramento Valley [25]: 28/14°C day/night temperatures, 50% relative humidity;natural light was supplemented by 900 µmol m^−2^ s^−1^ of photosynthetic photon flux density (PPFD) from metal halide and high pressure sodium lamps to maintain a 16-h day length; soluble fertilizer (Grow More, Inc., Gardena, CA) was applied through irrigation as needed. Seeds were harvested from panicles at the time of seed shattering in early fall, stored at 20°C for 3 weeks to approximate typical early autumn temperatures and thereafter stored at 3°C, approximating mid-winter temperatures. Water content of seeds kept in dry storage was 7 to 9% (dry weight basis).

### Effects of Dormancy on Germination

The role of dormancy in determining the range of environmental conditions that allow germination was assessed in spring 2008 in two concurrent experiments by comparing germination responses to gradients of water potential (*Ψ*) or oxygen concentration (*Ox*) in seeds that had been stratified (wet-chilled) for dormancy removal or not stratified (dry-chilled). Seeds of each population were subjected during 90 days to either stratification by 10 cm immersion in 500 ml water and storage at 3°C to simulate wintertime stratification [24] or to dry chilling at 3°C. Seeds were briefly surface sterilized by 0.5% NaOCl to reduce microbial growth during germination tests, but this did not affect dormancy without stratification. The *Ψ* and *Ox* experiments that followed were conducted in a growth chamber at a constant 25°C, as this falls within the range of optimal temperatures for germination in these populations [unpublished data], and 12 h day length under 200 µmol m^−2^ s^−1^ PPFD (halogen lights) to satisfy any light requirements for germination. Germinated seeds were counted and removed daily for the first 5 days of the experiment and every 2–3 days thereafter until day 24. Seeds were counted as germinated whenever coleoptile growth reached ≥ 1 mm. At the end of each experiment non-germinating seeds were tested for viability using tetrazolium [28].

#### Water potential experiment

Because of this species’ association with flooded environments [1], [2], a range of fairly moist conditions was simulated using polyethylene glycol 8000 (PEG, Fisher Scientific, Pittsburgh, PA) solutions prepared according to Michel [29] to create *Ψ* levels of 0 (pure DI water), −0.2, −0.4 and −0.7 MPa. These *Ψ* levels were verified with a dewpoint water potential meter (WP4 DewpointPotentiaMeter, DecagonDevices, Pullman, WA) at the beginning and end of the experiment. Experimental units were designed to prevent PEG or moisture loss through evaporation, and there was no change in *Ψ* levels over the period of germination. The experimental unit was a 14×14×5 cm transparent plastic container placed inside a 3.8 L airtight re-sealable clear plastic bag [16]. In each container, a set of 35 seeds for each combination of population and chilling treatment was attached to a 2.5 × 2.5 cm section of Velcro acrylic-based adhesive industrial strip (Uline, Pleasant Prairie, WI) that was fixed to the bottom surface, thus allowing seeds to remain in place 2 cm deep under 300 mL of either DI water or PEG solution per container. Pressurized 21% oxygen was humidified and equally distributed via a flow board through latex tubes (4.8 mm inner diameter, 9.5 mm outer diameter; Kent Elastomer Products, Inc., Kent, OH) at a rate of 600 mL min^−1^and bubbledinto containers [16]. Treatments were arranged in a split-plot factorial and replicated six times; *Ψ* levels were main plots and populations × chilling treatment were randomized sub-plots.

#### Oxygen concentration experiment

Using the same experimental arrangement described in the preceding section and following Al-Ani *et al*. [30], dormancy modification of germination responses to oxygen was evaluated using oxygen concentrations (*Ox)* of 21, 10, 1, 0.01 and 0.001% created using pressurized 21% O_2_ and premixed O_2_/N_2_ ratios (Airgas NCN, Sacramento, CA),and supplied to containers holding 300 mL of DI water. Upon being established in each container through an initial 5-minute flush of gas at a rate of 3000 mL min^−1^, *Ox* treatments were continuously distributed into containers as described above. Immediately before inflow into the containers, O_2_ concentrations in gas mixtures were verified with a headspace trace oxygen analyzer (Pac Check 650, Mocon Inc., Minneapolis, MN). Gas entered the containers via an airtight grommet-lined opening and emerged through an aquarium bubbler fixed to the bottom center of the container. Inflow of gas created a positive pressure inside the sealed bag that permanently pushed air outwards through a syringe needle [16]. Treatments were arranged in a split plot factorial and replicated six times; *Ox* levels were main plots and populations × chilling treatment were subplots randomized within.

#### Germination data analysis

Final germination was expressed as a percentage of the total number of seeds in a treatment.

The hydrotime population-based threshold model proposed by Gummerson [31] and described by Bradford [32] was used to quantify stratification effects upon germination responses to moisture stress. The model assumes that all seeds in a population require a constant hydrotime (*θ_H_*) for germination that is defined as:
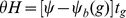
(1)


where *Ψ* is experimental water potential, *Ψ_b_*(*g*) is the base water potential below which germination is prevented for a given fraction *g* of the seed population, and *t_g_* is time to germination of *g*. The model also assumes *Ψ_b_* follows a normal distribution among seeds and is thus responsible for within-population variation in germination timing. Probit analysis was used to estimate the parameters of equation 1 [33]:
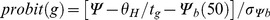
(2)where *Ψ_b_*(50) is median *Ψ_b_* and σ*_Ψb_* is the standard deviation in *Ψ_b_* among seeds in a population. We combined germination data from the range of experimental *Ψ* treatments and used equation 2 to predict and plot germination [34]. Following Huarte and Benech-Arnold [35], we used the Solver tool of Microsoft Excel (2003–2010) to derive parameters for this function by minimizing the root-mean-square error (RMSE) between simulated and observed data. ANOVA was conducted for each parameter and protected LSD (P<0.05) values obtained using JMP 8.0 software (SAS Institute Inc. Cary, NC); orthogonal contrasts of means were subsequently used to compare between R and S groups of populations.

Stratification effects on the germination response to decreasing oxygen were analyzed using an oxygen-time threshold model analogous to the hydrotyme model [13]. This model assumes a normal distribution of base oxygen threshold levels (*Ox_b_*) across the seed population and a constant oxygen time (*θ_Ox_*) to germination for all seeds. Germination responses are described as a function of the logarithm of the oxygen concentration:
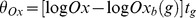
(3)where *Ox* is daily average oxygen percentage in the micro-environment surrounding the seed, *Ox_b_*(*g*) is the base or minimum level of oxygen just allowing germination of a given fraction *g* of seeds, and *t_g_* is time to germination for that same fraction. Parameter values for equation 3 were also derived from probit analysis:
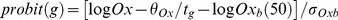
(4)where Oxb(50) is the median base oxygen level, and σΨbis the standard deviation of logOxb distribution among individual seeds in the population. Further data analysis was as described earlier for the hydrotime model.

### Dormancy Release Experiments

Three experiments evaluated the roles of temperature fluctuations and stratification conditions (temperature, moisture level, and duration) in releasing dormancy. Populations AM (S) and RD (R) [16], [27] were added to the previous set of four populations to better distinguish between R and S seeds. After harvest in fall 2009, seeds were stored dry at 20°C until March 2010, when they were surface-sterilized and exposed to dormancy-release treatments as described in the following sections. Upon completion of those treatments, sets of 50 seeds were removed from each dish, washed with 0.2% Captan fungicide for 1 minute, sprayed with 70% ethanol, rinsed with DI water for 1 minute, transferred to 3-cm Petri dishes containing 2 mL DI water, sealed with Parafilm, and placed in a growth chamber for germination. Germination conditions, set to approximate springtime conditions in the mid Sacramento Valley [25], were 14/26°C night/day, 14-h day length under 390 µmol m^−2^ s^−1^ PPFD, and 80% RH. Germinated seeds were counted and removed daily over two weeks.

#### Alternating temperatures

Sets of dry-stored non-chilled seeds, not exposed to any dormancy-releasing treatment, were placed in Petri dishes and simultaneously germinated at either constant 20°C or at alternating 14/26°C night/day using growth chambers set as described above. Four replicate sets of seeds per population were randomly distributed within each temperature regime.

#### Stratification temperature

Seeds of each population were placed in Parafilm-sealed Petri dishes containing 50 ml of DI water, wrapped in aluminum foil to control for light effects on dormancy release, and randomly arranged in covered 6.1 L plastic storage boxes in dark rooms set for constant 2.5, 5, and 7.5°C temperature regimes. These temperatures were selected because 5°C is the optimum chilling temperature for cold stratification in many species [24] and also because they all fall below the base temperature of approximately 9°C for germination in these populations [16], thus avoiding confounding effects of dormancy release and germination. After 24 days of stratification, seeds were transferred to a growth chamber for germination, as described above. Three replicates per population were arranged in a completely randomized design at each temperature.

#### Stratification duration and moisture stress

Seeds of each populationwere placed in Parafilm-sealed Petri dishes containing 50 mL PEG solutions of either 0 (DI water), −0.4, −0.8 or −1.6 MPa and kept under a constant 5°C for either 0, 3, 4, 7, 10, 14, 17, 28 , 35, 57 or 92 days. The *Ψ* spanned the range from no water stress to stress below −1.5 MPa, which is the permanent wilting point of many herbaceous species [36]. The *Ψ* levels were established and verified as described earlier, andwereverified every three weeks. Upon completion of stratification treatments, seeds were germinated as described earlier. Treatments were arranged in a split-plot factorial where population x *Ψ* combinations were in main plots and stratification durations were in sub-plots; there were two replicate sets of seeds in a completely randomized design.

#### Dormancy release data analysis

Dormancy-releasing effects of alternating temperatures and of stratification temperature, moisture stress and stratification duration were assessed by comparing germination rates (GR). The GR for a given treatment was calculated as the inverse time to median germination (i.e, 50% of the total seed population), which was estimated for each blocked replicate by fitting cumulative germination data with a three-parameter log-logistic equation using the R statistical program with the *drc* add-on package [37]:
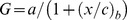
(5)where *G* is cumulative germination, *a* is the upper asymptote (the lower asymptote is assumed to be 0), *x* is days, *c* is time to 50% germination and *b* is the slope. The inverse of the thus calculated *c* parameter equals the GR [32]. Final germination (% of total seeds germinated) and GR were subjected to ANOVA of Box-Cox transformed data (when required to meet assumptions of homogeneity of variance). Orthogonal contrasts between means were subsequently used to compare between R and S groups of populations. All treatments achieved complete final germination, thus equation 5 yielded an accurate estimate of the median germination time for the population.Germination rates (GR) were used for comparisons among treatments as they vary linearly with temperature and *Ψ*, whereas median times to germination vary non-linearly with respect to these factors; while germination tests can be done at warmer temperatures, germination time course at all suboptimal temperatures can be predicted based on the GR and base temperatures [19].

## Results

### Effects of dormancy on germination

**Figure 1:**
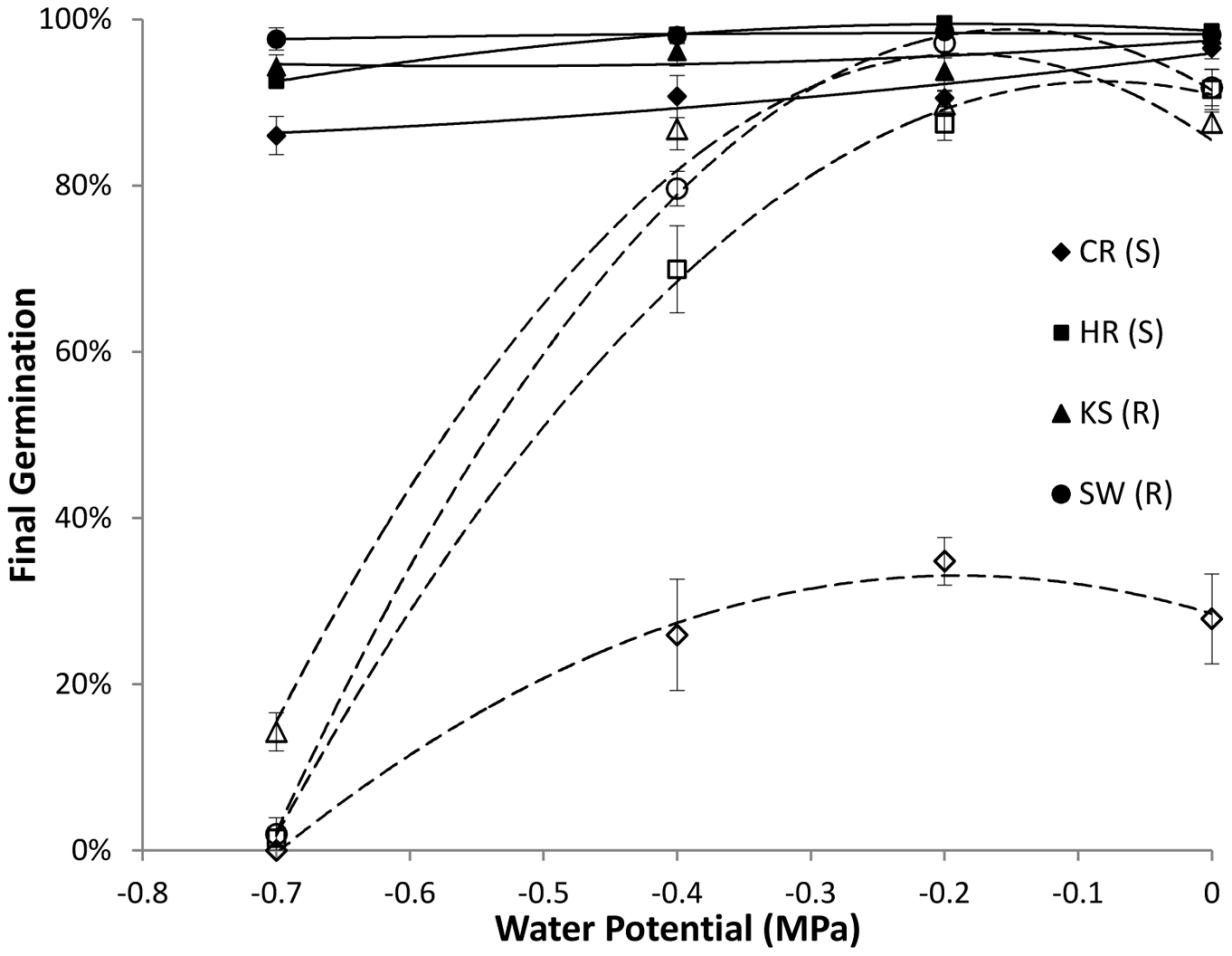
Final germination of stratified (solid lines and symbols) and nonstratified (dashed lines, open symbols) seeds of herbicide-resistant (R) populations (KS and SW) and –susceptible (S) *E. oryzicola* populations (CR and HR) across a range of water potentials. Seeds were germinated for 14 days at 25°C and 21% oxygen following three months of chilling at 3°C. Symbols represent averages of 6 observations ±SE; the LSD_0.05_ for the interaction between population and stratification treatment was 7% with 192 d.f.; lines are polynomial regressions.

**Figure 2:**
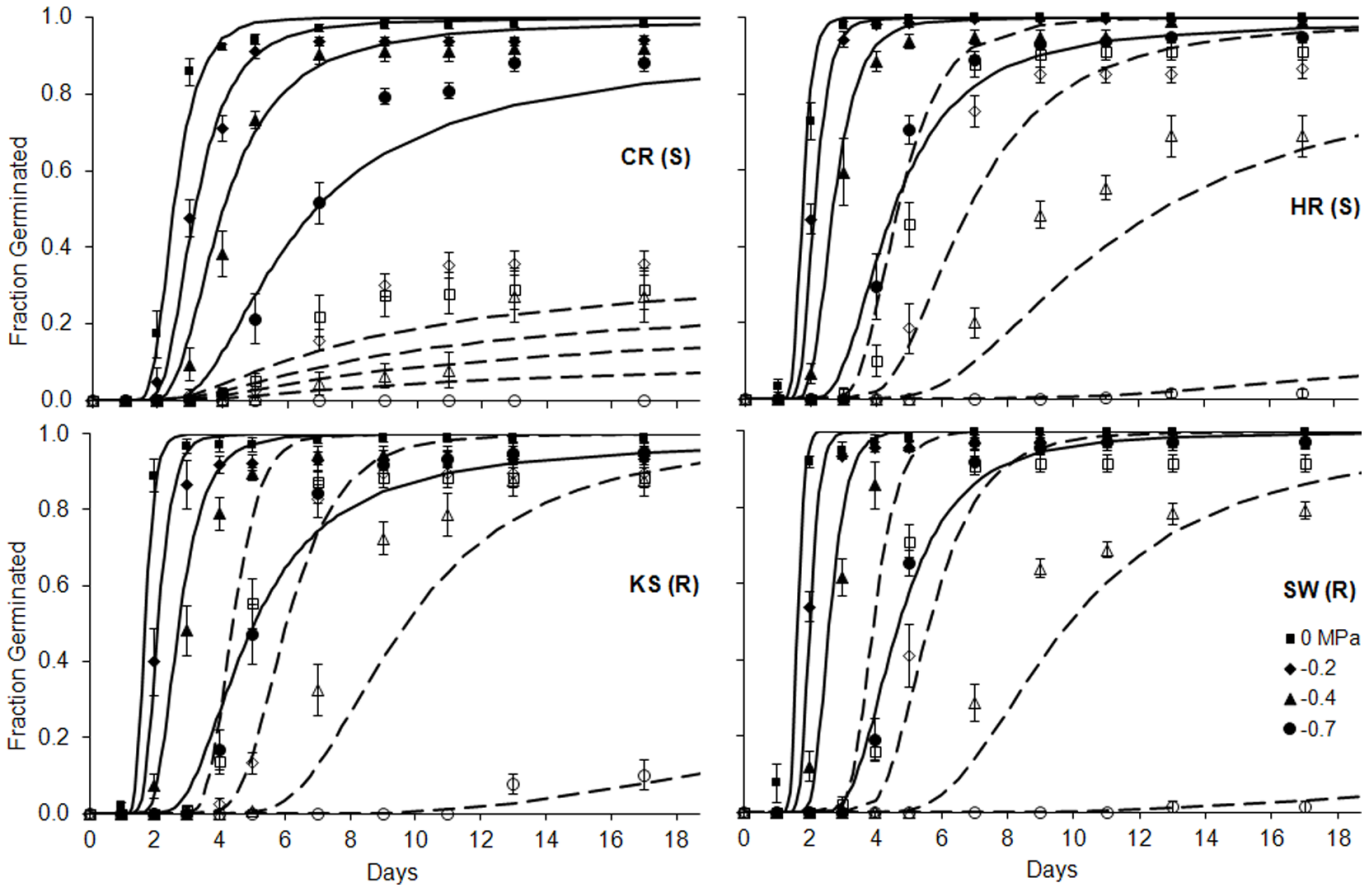
Average observed (symbols) and predicted (lines) germination among herbicide-resistant (R) and –susceptible (S) *E. oryzicola* populations across a gradient of for stratified (solid) and nonstratified (open/dashed) seeds. Seeds were germinated following three months of chilling at 3°C in water (stratified) or under dry conditions (nonstratified). Hydrotime germination models were fit by replicate with the equation* probit(g)*  =  [*Ψ* – *θ_H_*/*t_g_*–*Ψ_b_*(50)]/σ*_Ψb_*, where *Ψ* is experimental water potential, *θ_H_* is the hydrotime constant to germination, *t_g_* is time to germination of fraction *g* of the seed population, *Ψ_b_*(50) is median base water potential and σ*_Ψb_* is the standard deviation in *Ψ_b_* among seeds in a population. Average stratified and nonstratified root mean squared errors (RMSE) ±SE were: 0.079±0.009 and 0.082±0.009 for CR, 0.065±0.006 and 0.107±0.003 for HR, 0.082±0.005 and 0.116±0.014 for KS, and 0.077±0.006 and 0.126±0.007 for SW, respectively. Symbols represent averages of observations and bars represent SE based on six replicate sets of 35 seeds.

All populations germinated to high percentages (85–90%) across *Ψ* when stratified (Figure 1). However, final germination of nonstratified seeds approached 0% as *Ψ* decreased to −0.7 MPa, and was notably low for the S population CR. Tetrazolium testing of nonstratified seeds that failed to germinate at −0.7 MPa indicated generallyhigh seed viability across populations: 91±3, 97±2, 65±10 and 92±3% for CR, HR, KS and SW, respectively. Dormancy release by stratification enabled faster germination and under drier conditions (Figure 2) by reducing average *θ_H_* by 38% and decreasing *Ψ_b_*(50) compared to nonstratified seeds (Table 1). Stratification only improved germination synchrony (σ*_Ψ_*_b_) for the CR population. The hydrotime model fitted better the germination of stratified seeds, as indicated by the lower RMSE values (Figure 2). Model parameters suggested no consistent differences between the R and S seeds in their germination time course responses to moisture availability (Table 1). The two S populations differed from each other (p<0.001) in all three hydrotime model parameters, while differences between the two R populations were not significant.

**Table 1:** Parameters of the hydrotime model (Equation 2) characterizing the responses of herbicide-resistant (R) and –susceptible (S) *E. oryzicola* populations germinated at 25°C, 21% oxygen and *Ψ* of 0, −0.2, −0.4 or −0.7 MPa, after being stratified [16] or not for three months at 3°C; *θ_H_* is the hydrotime constant; *Ψ_b_*(50) is median base water potential, and*σ_Ψb_* is the standard deviation in *Ψ_b_* among seeds around *Ψ_b_*(50).

*E. oryzicola*	*θ_H_*	*Ψ_b_*(50)	*σ_ψb_*
Population	(MPa d^−1^ ±SE)	(MPa ±SE)	(MPa ±SE)
*Stratified*			
CR (S)	2.91±0.13	−1.12±0.03	0.27±0.02
HR (S)	1.97±0.21	−1.13±0.03	0.17±0.07
KS (R)	1.81±0.10	−1.06±0.04	0.15±0.04
SW (R)	1.75±0.04	−1.07±0.01	0.11±0.02
Average	2.11	−1.10	0.18
*Nonstratified*			
CR (S)	4.74±0.90	0.27±0.19	0.84±0.22
HR (S)	2.94±0.14	−0.63±0.02	0.15±0.01
KS (R)	3.22±0.12	−0.73±0.02	0.11±0.01
SW (R)	2.62±0.15	−0.66±0.02	0.10±0.00
Average	3.38	−0.37	0.30
*LSD_0.05_* (error d.f. = 48)			
Population (A)	0.23		
Stratification (B)	0.32		
A × B	NS	0.15	0.09

Models were fit for each of six replicates; values are parameter averages ±SE.

**Figure 3:**
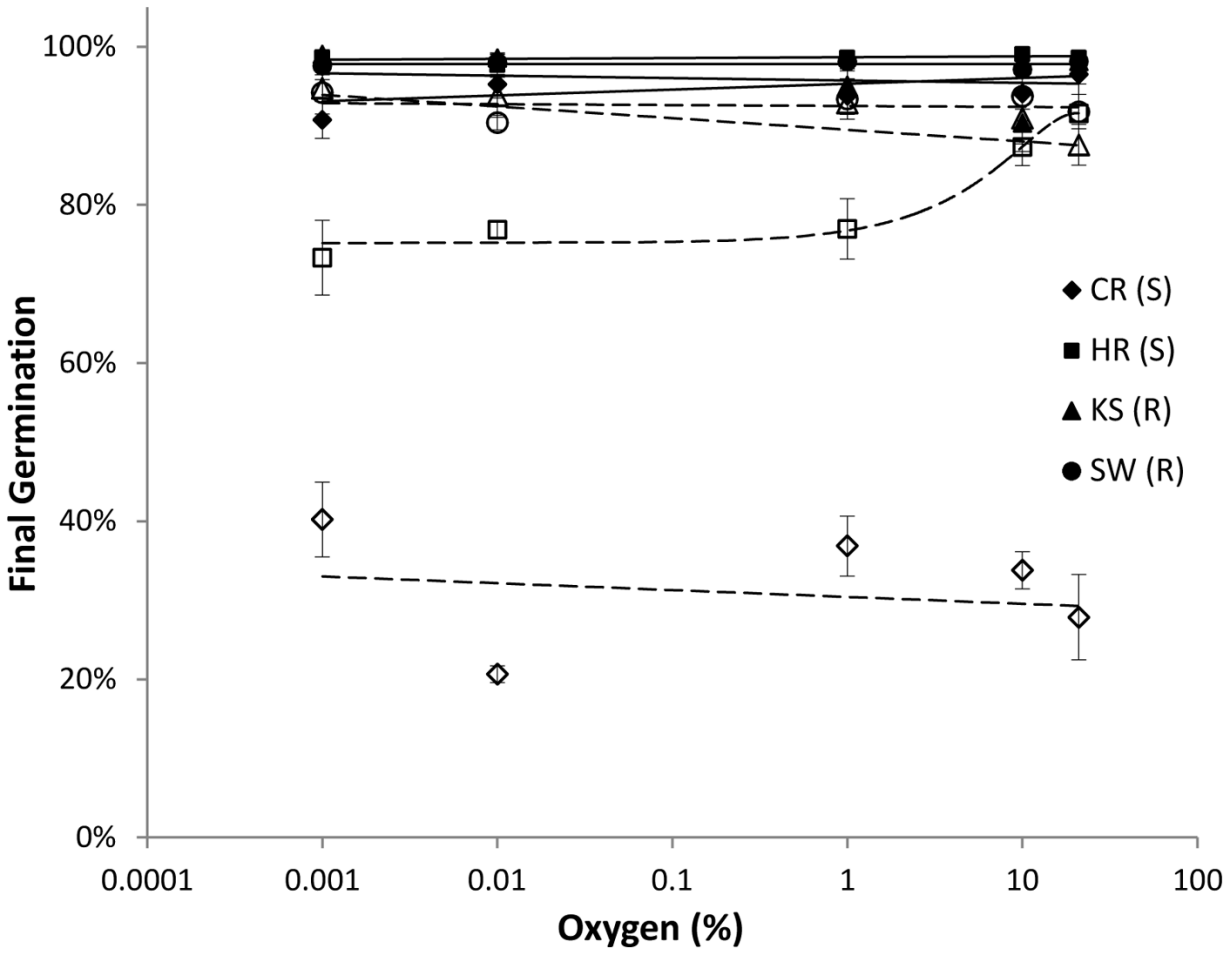
Final germination of stratified (solid lines and symbols) and nonstratified (dashed, open symbols) seeds of herbicide-resistant (R) and –susceptible (S) *E. oryzicola* populations across a range of oxygen levels. Seeds were germinated at 25°C and 0 MPa, following three months of chilling at 3°C in water (stratified) or under dry conditions (nonstratified). Symbols represent averages of observations ±SE based on six replicate sets of 35 seeds; lines are linear regressions. The LSD_0.05_ for the interaction between population and stratification treatment was  = 7% with 192 d.f.

**Figure 4:**
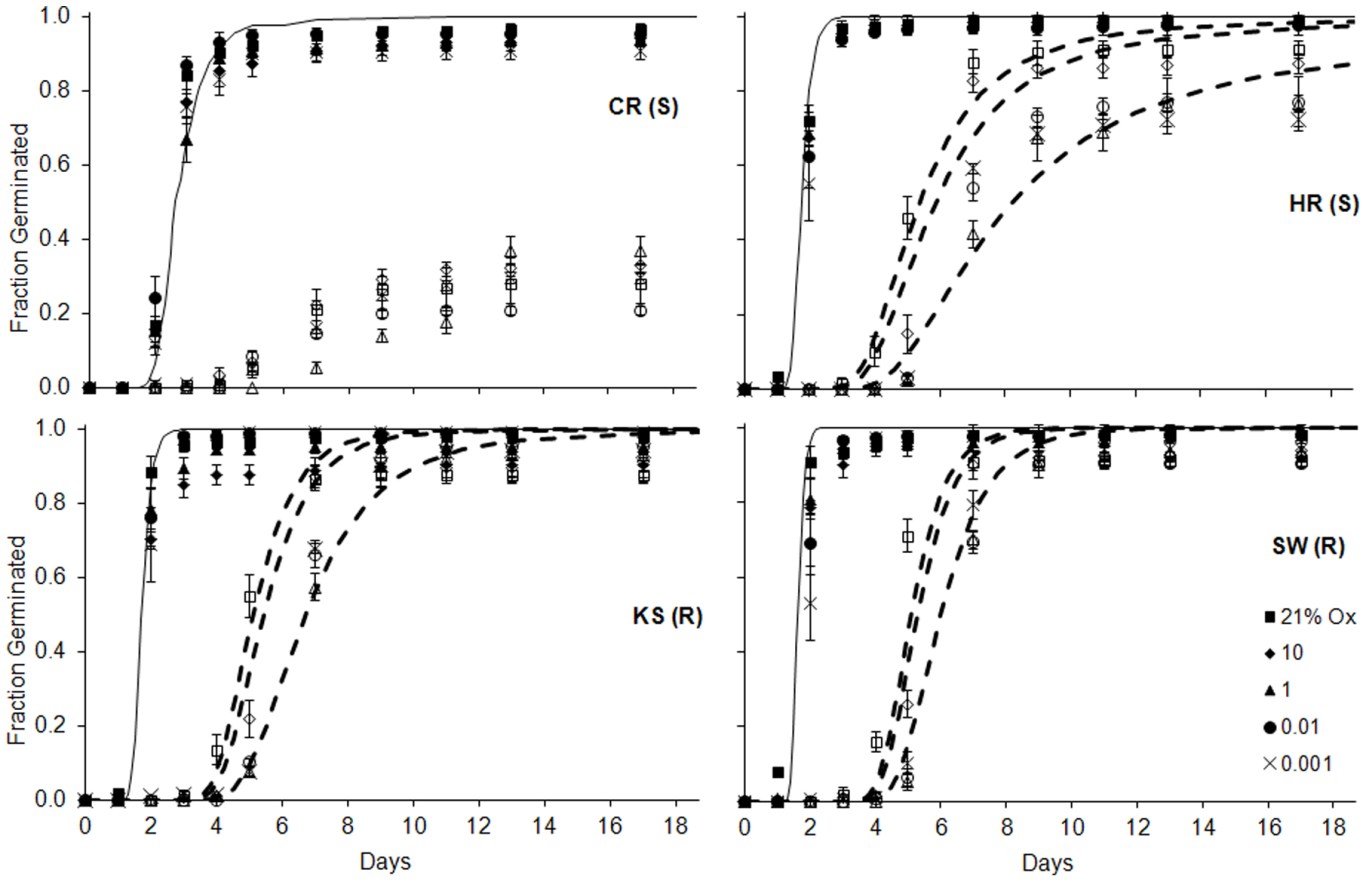
Average observed (symbols) and predicted (lines) germination among herbicide-resistant (R) and –susceptible (S) *E. oryzicola* populations across a gradient of oxygen levels (*Ox*) in stratified (solid) and nonstratified (open/dashed) seeds. Modeling of oxygen time was only applied when an oxygen dose response was observed (nonstratified seeds in the 21-1% *Ox* range) by fitting to each replicate the equation *probit(g)*  =  [log*Ox* – *θ_Ox_*/*t_g_*–log*Ox_b_*(50)]/σ*_Oxb_* where *Ox* is daily average oxygen percentage in the micro-environment surrounding the seed, *θ_Ox_* is the oxygen time constant, *t_g_* is time to germination for fraction *g* of the seed population, *Ox*_b_(50) is the median base oxygen level, and σ*_Ψb_* is the standard deviation of the log*Ox_b_* distribution among individual seeds in the population.Average RMSE ±SE for nonstratified seeds were 0.084±0.003, 0.075±0.010; and 0.077±0.011 for HR, KS and SW, respectively. A hydrotime model for 0 MPa (Equation 2) was fit to data from germination time-courses of stratified seeds; when compared to observed germination across *Ox*, RMSE for these models were 0.096±0.005, 0.093±0.010, 0.087±0.012 and 0.128±0.016 for CR, HR, KS and SW, respectively. Symbols represent averages of observations and bars represent SE based on six replicate sets of 35 seeds.

Stratified seeds were insensitive to changes in oxygen availability (Figures 3–4) even at levels as low as those of flooded paddy fields [38]. Stratification enhanced final germination of S seeds only (Figure 3). Thus germination of nonstratified HR seeds decreased by 15% when *Ox* dropped to 1%, andfinal germination of nonstratified CR seeds was notably low and insensitive to oxygen availability (Figure 3). Tetrazolium testing confirmed viability of non-germinating HR and CR seeds (on average 72±6 % and 72±12% across *Ox* for HR and CR, respectively). Germination time courses of stratified *E. oryzicola* seeds in all populations were almost insensitive to hypoxia and sensitivity was even limited in nonstratified seeds (Figure 4), which had an average*Ox_b_*(50) of 0.04 ppm (not shown). Given this general lack of responses, an oxygen threshold model could only be fit to nonstratified seeds of three of the populations germinating under *Ox* of 1 to 21%, but model parameters still reflected only minor sensitivity to *Ox* and were not statistically different across populations (not shown). This minor sensitivity disappeared under stratification and germination rates were faster than for non-stratified seeds under all *Ox* levels (Figure 4).

### Dormancy release

#### Effect of alternating temperatures

Full final germination was achieved by all populations under both constant and alternating temperatures, but alternating temperatures nearly doubled germination rates (GR) in all populations (Table 2). Average GR for R populations (KS, RD, SW) were greater (p<0.001) than for S populations under both constant (0.15±0.00 vs. 0.13±0.00, respectively) and alternating (0.24±0.00 vs. 0.20±0.01, respectively) temperature regimes.

**Table 2:** Effects of alternating temperatures on final germination (G) and germination rates (GR, calculated by replicate as the inverse of median time to germination (*1/t_50_*) using Equation 5) of herbicide-resistant (R) and –susceptible (S) *E. oryzicola*.

	Constant Temperature		Alternating Temperature	
*E. oryzicola* Population	Final G(%±SE)	GR (1/t_50_ ±SE)	Final G(%±SE)	GR(1/t_50_ ±SE)
AM (S)	98±3	0.12±0.00	100±0	0.19±0.01
CR (S)	94±3	0.11±0.02	100±0	0.19±0.01
HR (S)	98±2	0.14±0.00	100±0	0.21±0.01
KS (R)	100±0	0.15±0.00	100±0	0.23±0.00
RD (R)	100±0	0.16±0.00	100±0	0.25±0.01
SW (R)	100±0	0.15±0.00	100±0	0.25±0.01
Average	98	0.13	100	0.22
*LSD_0.05_* (error d.f. = 24)				
Final G			2.2	
GR Population x Temperature regime			0.01	

Nonstratified seeds were germinated at 0 MPa and 21% oxygen, with temperatures either held constant at 20°C or set to 14/26°C day/night regime. Values are averages ±SE of four replicate sets of 50 seeds.

#### Decreasing stratification temperature

Germination rates were similar at stratification temperatures of 2.5 and 5°C, but increased when temperatures rose from 5 to 7.5°C(Table 3). There were no differences (p = 0.890) in GR among R and S *E. oryzicola* populations within the range of stratification temperatures studied (Table 3).

**Table 3:** Germination rates (GR, calculated by replicate as the inverse of median time to germination (*1/t_50_*) using Equation 5) following 24 days of stratification at three temperatures <*T_b_* for germination.

*E. oryzicola*	Temperature regime		
Population	2.5°C	5°C	7.5°C
		*GR (1/t_50_)*	
AM (S)	0.67±0.04	0.67±0.02	0.78±0.04
CR (S)	0.56±0.02	0.62±0.02	0.74±0.06
HR (S)	0.60±0.06	0.65±0.04	0.85±0.09
KS (R)	0.66±0.05	0.67±0.04	0.81±0.01
RD (R)	0.64±0.03	0.61±0.02	0.74±0.09
SW (R)	0.66±0.03	0.60±0.01	0.79±0.05
Average	0.63	0.64	0.79
*LSD_0.05_*(d.f. = 18)	NS	NS	NS

Herbicide-resistant (R) and –susceptible (S) *E. oryzicola* seeds were germinated at 14/26°C night/day, 0 MPa and 21% oxygen. Values are averages ±SE of 3 replicate sets of 50 seeds.

#### Stratification duration and moisture stress

**Figure 5:**
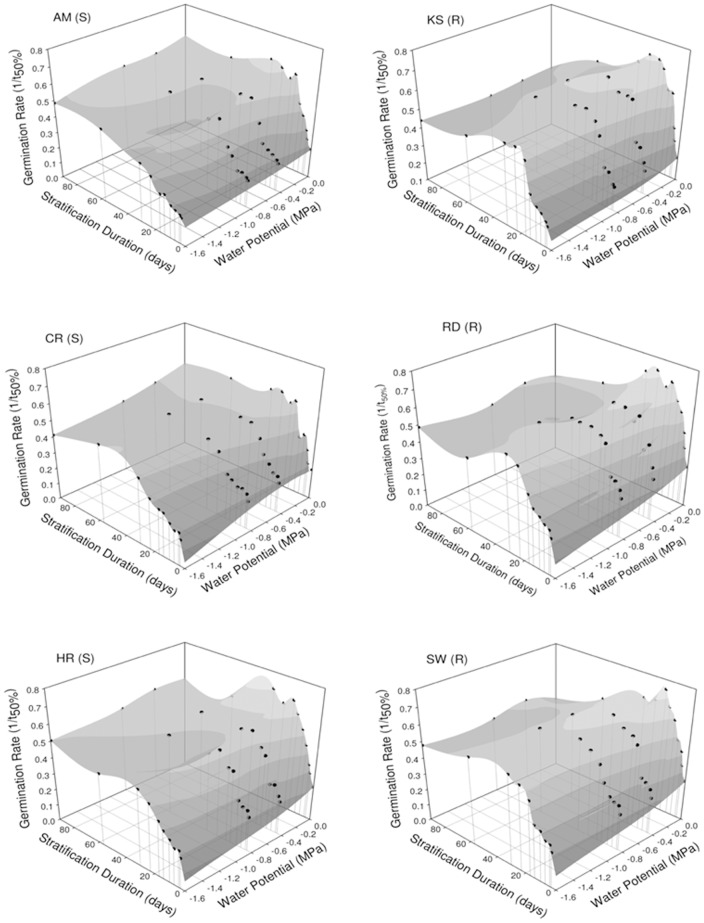
Herbicide-resistant (R) and –susceptible (S) *E. oryzicola* germination in response to stratification duration and water potential. Seeds were immersed in PEG solutions of 0, −0.4, −0.8 and −1.6 MPa at a constant 5°C for 3, 4, 7, 10, 14, 17, 23, 30, 57 and 92 days prior to germination at 21°C and 0 MPa. Final germination for all treatments was ≥95%. Germination rates were calculated by replicate as the inverse of median time to germination (*1/t_50_*), which was determined from Equation 5. Seeds were germinated at 17/24°C night/day temperatures, 0 MPa and 21% oxygen. Symbols are averages of two replicates of 50 seeds per treatment. Peak GR was attained at 30, 23, 30, 23, 23 and 17 days of stratification in 0 MPa for AL, CR, HR, KS, RD and SW, respectively. The LSD_0.05_ for the interaction between population, stratification duration and stratification *Ψ* was 0.06; d.f.  = 478.

Final germination percentages were consistently ≥ 95% (not shown) and the enhancement of GR due to stratification varied (p <0.05) with the duration of wet-chilling and moisture stress during that period (Figure 5). Thus GR peaked after 17–30 days of immersion in water (*Ψ* = 0 MPa), and maximum GR values declined with *Ψ*<0 MPa(Table 4). Lengthening stratification duration did not mitigate decreases in GR as *Ψ* became more negative (Figure 5). Average GR was higher for R populations than for S populations (0.46±0.01 and 0.39±0.01, respectively; p<0.001) across stratification durations and *Ψ* levels. Stratification in water beyond the time to maximum GR ultimately led to greater reductions in GR for the R than for the S populations (Figure 5).

**Table 4:** Maximum germination rates (GR, calculated by replicate as the inverse of median time to germination (*1/t_50_*) using Equation 5) after stratification at 5°C under various water potentials.

*E. oryzicola*	Water Potential				
Population	0 MPa	−0.4 MPa	−0.8 MPa	−1.6 MPa	Average
	*GR (1/t_50_)* ±SE				
AM (S)	0.67±0.04	0.56±0.01	0.55±0.02	0.48±0.01	0.57±0.03
CR (S)	0.59±0.02	0.51±0.01	0.50±0.01	0.46±0.03	0.52±0.02
HR (S)	0.71±0.00	0.59±0.01	0.54±0.01	0.52±0.01	0.59±0.03
KS (R)	0.72±0.01	0.66±0.05	0.58±0.03	0.55±0.01	0.63±0.03
RD (R)	0.77±0.07	0.61±0.01	0.57±0.01	0.56±0.03	0.64±0.03
SW (R)	0.78±0.08	0.66±0.05	0.58±0.02	0.57±0.02	0.64±0.04
Average	0.71±0.02	0.60±0.02	0.55±0.01	0.52±0.01	
*LSD_0.05_* (error d.f. = 48)^a^					
Population (A)		0.03			
Water Potential (B)		0.03			

GR was assessed for each herbicide-resistant (R) and –susceptible (S) *E. oryzicola* population after 0, 3, 4, 7, 10, 14, 17, 28, 35, 57 and 92 days of stratification and values are averages ±SE of the maximum GR for each replicate.

a. To meet ANOVA assumptions, a Box-Cox transformation (λ  =  −1.8) was applied.

## Discussion

### Effects of stratification on germination responses to moisture and oxygen

Reductions in both *θ_H_* and *Ψ_b_*(50)indicated dormancy removal and broadened the range of water potentials at which germination could occur following stratification [39], [40]. Thus dormancy was partly associated with reduced ability to germinate under drier conditions, which is a logical adaptive trait for an aquatic species like *E. oryzicola* [1]. This was further reflected in the dramatic drop in germination of nonstratified seeds when *Ψ* fell to −0.7 MPa (Figure 1), and in the positive *Ψ_b_*(50) that reflected the inability of nonstratified CR seeds to germinate(Table 1, Figure 1). The overall reduction in*Ψ_b_*(50) due to stratification is consistent with similar reductions from after-ripening in red rice [41] and stratification in *Polygonum* [40]. Poorer hydrotime model predictions of germination time courses for nonstratified seeds (Figure 2) suggest sensitivity to other dormancy-related variables, such aslight, nitrate or hormone levels, which may complicate the application of this model when dormancy is present in a population [42].

A transition from aerobic respiration to anaerobic metabolism enables adaptation of this weed to the flooding of paddy fields, and previous research has linked dormancy removal in *E.oryzicola*to increased ability to germinate under hypoxia through anaerobic fermentation [1]. In our study, stratification removed a trend towards slower germination at the lower *Ox* levels in three nonstratified populations (HR, KS and SW) (Figure 4). Nevertheless, the range of germination responses to *Ox*levels for the nonstratified seeds of these three populations was narrow and the estimated *Ox_b_*(50) values (∼ 0.04 ppm) fell well below those of most other species [13]. Thus *E. oryzicola* exhibited limited sensitivity to hypoxia consistent with the known ability of this weed to germinate under flooded conditions [1]. The somewhat lower final germination of nonstratified HR (S) seeds at reduced *Ox*levels (Figure 3) could suggest that a fraction of that seed population had comparatively greater requirements of aerobic respiration to germinate [13]. The timing of the *E. oryzicola* transition from aerobic to fermentative metabolism and its relationship to stratification and dormancy removal requires further study. The lower final germination of nonstratified CR (S) seedsacross *Ox* levels (Figure 3) could be explained bytheir positive *Ψ_b_*(50) value (Table 1) rather than as an oxygen response.

Stronger similarities between the R populations KS and SW than between the S populations CR and HR in hydrotime model parameters (Table 1) and in final germination across *Ψ* and *Ox*levels (Figures 1 and 3) corroborate previous reports of reduced phenotypic variation among R populations compared to S [23], [27].

### Dormancy release by alternating temperatures

Dormancy release by alternating temperatures typical of springtime at the time of rice seeding in the Sacramento Valley of California [25] was expressed as an increase in GR for seeds of all populations used in these experiments, rather than by a change in final germination percentage (Table 2). These observations are consistent with previous reports that fluctuating temperatures aid in alleviating dormancy [43]–[45], and indicate that dormancy was present in our seeds [46]. Thus our results corroborate previous reports on the presence of seed dormancy in *E. oryzicola* [1]. However, as seeds were stored at room temperature for a few months prior to experimentation and NDPD release can already begin during dry after-ripening [47]–[49], some loss of dormancy could already have occurred.

### Dormancy release by stratification at different temperatures and *Ψ*

Dormancy release by stratification was expressed primarily through increases in germination rates.Studies with other species have documented enhancement of stratification effects as stratification temperatures were reduced below *T_b_*: such a relationship has been the basis for the application of population-based stratification-time models [50], [51]. However, GR in our seeds was not enhanced by lowering stratification temperatures (Table 3) but, instead, by raising them to 7.5, which is close to the *T_b_*(50) for *E. oryzicola* [16]. Further experimentation would be required to define whether this increase in GR limited to a narrow temperature range signifies a the beginning of a trend toward enhanced dormancy release with rising temperatures, or the onset of germination, or both.

In California rice growing areas, winter temperatures will remain within 5°C of *T*_b_ for *E. oryzicola* germination [25], but the degree of wintertime chilling is beyond the control of land managers. However, growers may modify the hydric status of their fields through irrigation; thus our research also focused on the effects of moisture on dormancy release.We found lower maximum GR values following stratification under decreasing *Ψ* (Table 4),which impliesthat dormancy release may be in part controlled by winter moisture levels in this wetland species. Stratification duration to full dormancy removal, as represented by maximum GR in these populations (Figure 5), was less than that reported for *Polygonumaviculare* [40], *Bromustectorum* [47] and for weedy rice [41], suggesting dormancy is either lower or more readily removed in our *E. oryzicola* populations.

Since seeds used for the germination studies were stratified for 90 days, some measure of secondary dormancy may have been induced [51]–[53]. However, the large differences in observed germination responses between stratified and nonstratified seeds are evidence that significant levels of dormancy had been removed by stratification. Secondary dormancy can lead *E. oryzicola* seeds to persist in seedbanks for as many as ten years [1]; thus although weed control techniques for this weed can be successful [8], [9], the complete eradication of the weed from a rice field is difficult [1].

Applications of this research to field conditions must take into account that dormancy levels may have been influenced to varying degrees by each of the general experimental conditions to which seeds were subjected [24]. These include environmental factors, such as fixed temperature regimes and photoperiods, experienced by seeds during their development and maturation [21], [24], [54], as well as seed cleaning procedures and storage conditions after harvest but before experiments began [20], [24], [55], [56].Post-harvest seed storage temperatures may have influenced dormancy levels, as could the length of storage prior to experimentation.Indeed, there is evidence that*Arabidopsis*germination may be more affected by the postdispersal environment than by certain conditions affecting seed maturation [57]; however,such results were not explicitly tied to dormancy norto grasses. Therefore, any inferences made to field conditions should be adequately tested through field experiments.

Winter flooding is often practiced by California rice growers to facilitate stubble decomposition [26],a practice that may help *E. oryzicola* control by hastening its springtime GR,providedwintertime soil saturation can be maintained for an extended period of time (Figure 5). Herbicide-resistant populations generally exhibited higher germination rates under either constant temperatures (Table 2) and when stratified at sub-optimal *Ψ* (<0 MPa), suggesting lower dormancy levels. Greater basal ethylene levels, which can play a causal role in the metabolic response to submergence [58], have been detected in R plants compared to S plants [59]. Since increased ethylene has been shown to correlate to increased dormancy release and germination in some species [60], further research might explore whether higher basal ethylene levels in R seeds might contribute to their higher GR.

In summary, *E. oryzicola* seed dormancy was manifested primarily by reduced GR rather than by lower final germination percentages, and was released by alternating temperatures and by stratification. Stratification led to dormancy release characterized by hastened germination rates of unstressed seeds, enhanced ability to accrue hydrotime and germinate under drier conditions, and a minor increment of the ability to germinate under hypoxia. There was, otherwise, little germination response to oxygen availability in the *E. oryzicola* seeds tested, consistent with the wetland habit of this weed. The effects of stratification on GR were generally more sensitive to changes in *Ψ* than to temperature. Dormancy levels may have been lower among R populations compared to S populations in these experiments,but R seeds may also be more prone to secondary dormancy induction as stratification duration increases. A weed seed bank depletion program based on favoring weed emergence for subsequent control, such as with the stale seedbed technique, would benefit from optimizing environmentalconditions for weed seed germination. Based on our results, and depending on the degree of dormancy of the population, *E. oryzicola* dormancy release to enhance weed seed germination would benefit from field soil saturation in winter. Thiswill decrease time to seedling emergence,allowing for early-season weed control and a shortened crop planting delay.
